# The potential role of chemotaxis and the complement system in the formation and progression of thoracic aortic aneurysms inferred from the weighted gene coexpression network analysis

**DOI:** 10.1186/s12967-021-02716-6

**Published:** 2021-02-02

**Authors:** Chuxiang Lei, Dan Yang, Wenlin Chen, Haoxuan Kan, Fang Xu, Hui Zhang, Wei Wang, Lei Ji, Yuehong Zheng

**Affiliations:** 1 Department of Vascular Surgery, Peking Union Medical College Hospital, Peking Union Medical College and Chinese Academy of Medical Sciences, No 1. Shuaifuyuan, Dongcheng District, Beijing, China; 2 Department of Computational Biology and Bioinformatics, Institute of Medicinal Plant Development, Chinese Academy of Medical Sciences and Peking Union Medical College, Beijing, China; 3 Department of Neurosurgery, Peking Union Medical College Hospital, Chinese Academy of Medical Sciences and Peking Union Medical College, Beijing, China

**Keywords:** Thoracic aortic aneurysm, Weighted gene coexpression network analysis, Chemotaxis, Immune infiltration

## Abstract

**Background:**

Thoracic aortic aneurysm (TAA) can be life-threatening due to the progressive weakening and dilatation of the aortic wall. Once the aortic wall has ruptured, no effective pharmaceutical therapies are available. However, studies on TAA at the gene expression level are limited. Our study aimed to identify the driver genes and critical pathways of TAA through gene coexpression networks.

**Methods:**

We analyzed the genetic data of TAA patients from a public database by weighted gene coexpression network analysis (WGCNA). Modules with clinical significance were identified, and the differentially expressed genes (DEGs) were intersected with the genes in these modules. Gene Ontology and pathway enrichment analyses were performed. Finally, hub genes that might be driving factors of TAA were identified. Furthermore, we evaluated the diagnostic accuracy of these genes and analyzed the composition of immune cells using the CIBERSORT algorithm.

**Results:**

We identified 256 DEGs and two modules with clinical significance. The immune response, including leukocyte adhesion, mononuclear cell proliferation and T cell activation, was identified by functional enrichment analysis. *CX3CR1*, *C3*, and *C3AR1* were the top 3 hub genes in the module correlated with TAA, and the areas under the curve (AUCs) by receiver operating characteristic (ROC) analysis of all the hub genes exceeded 0.7. Finally, we found that the proportions of infiltrating immune cells in TAA and normal tissues were different, especially in terms of macrophages and natural killer (NK) cells.

**Conclusion:**

Chemotaxis and the complement system were identified as crucial pathways in TAA, and macrophages with interactive immune cells may regulate this pathological process.

## Introduction

Aortic aneurysm is a common vascular disease defined as aortic enlargement or expansion with an artery diameter > 1.5 times the average [1]. Thoracic aortic aneurysm (TAA), characterized by a weakening and dilatation of the ascending aorta, is usually asymptomatic until dissection. TAA can be a potentially life-threatening condition due to rupture because of its expansion and progression characteristics. Abdominal aortic aneurysm (AAA), another aortic aneurysmal disease with an incidence rate of up to 1% ~ 2% among men aged ≥ 65 years [2], shares several but limited common clinical and pathological characteristics with TAA [3]. In contrast to AAA driven primarily by atherosclerosis, genetic influences play a more prominent role in the occurrence and development of TAA [4, 5]. However, there are fewer previous studies related to the pathological mechanisms of TAA than AAA, regardless of the notable differences between these two diseases.

Similar to AAA, TAA is characterized by impairment of aortic structural integrity due to abnormalities in the extracellular matrix. The related molecules and mechanisms include genetic variants [6, 7], apoptosis or necroptosis of vascular smooth muscle cells (VSMCs), matrix metalloproteinases (MMPs) [8], inflammation [9], and reactive oxygen species (ROS) [10, 11]. Previous studies have reported the cardinal factor of inflammation in AAA, while the evidence supporting the inflammatory role in TAA is less extensive [12]. However, several inflammatory and immune cells, including T cells and macrophages, were observed in human TAA, and multiple relative inflammatory pathways were upregulated [9, 13]. Studies on a murine TAA model found elevated interleukin-1β (IL-1β) and interleukin-6 (IL-6) levels compared to those in normal tissues. Interestingly, IL-6 levels were also significantly higher in both murine and human TAA than in AAA [14, 15]. These data suggest the crucial role of immune and inflammatory processes in TAA and the differences between the mechanisms of TAA and AAA.

With the technological advances of microarray, high-throughput sequencing and bioinformatic analyses, we are now able to detect transcriptome changes and molecular mechanisms in many diseases. Several studies have investigated the molecular mechanisms of TAA formation and development at the level of differentially expressed genes (DEGs). Although these DEGs and related pathways might participate in TAA, the exact complex networks and interactions among crucial genes and proteins that drive the disease are virtually unknown. In this study, weighted gene coexpression network analysis (WGCNA) was performed on the microarray data of TAA patients and normal aorta from a public database to identify significant modules that were closely related with TAA. It may provide a better understanding of the molecular mechanisms of TAA from a new perspective.

## Materials and methods

The data and the codes that support the findings of this study are available from the corresponding author upon reasonable request.

### Study design and data collection

**Fig. 1:**
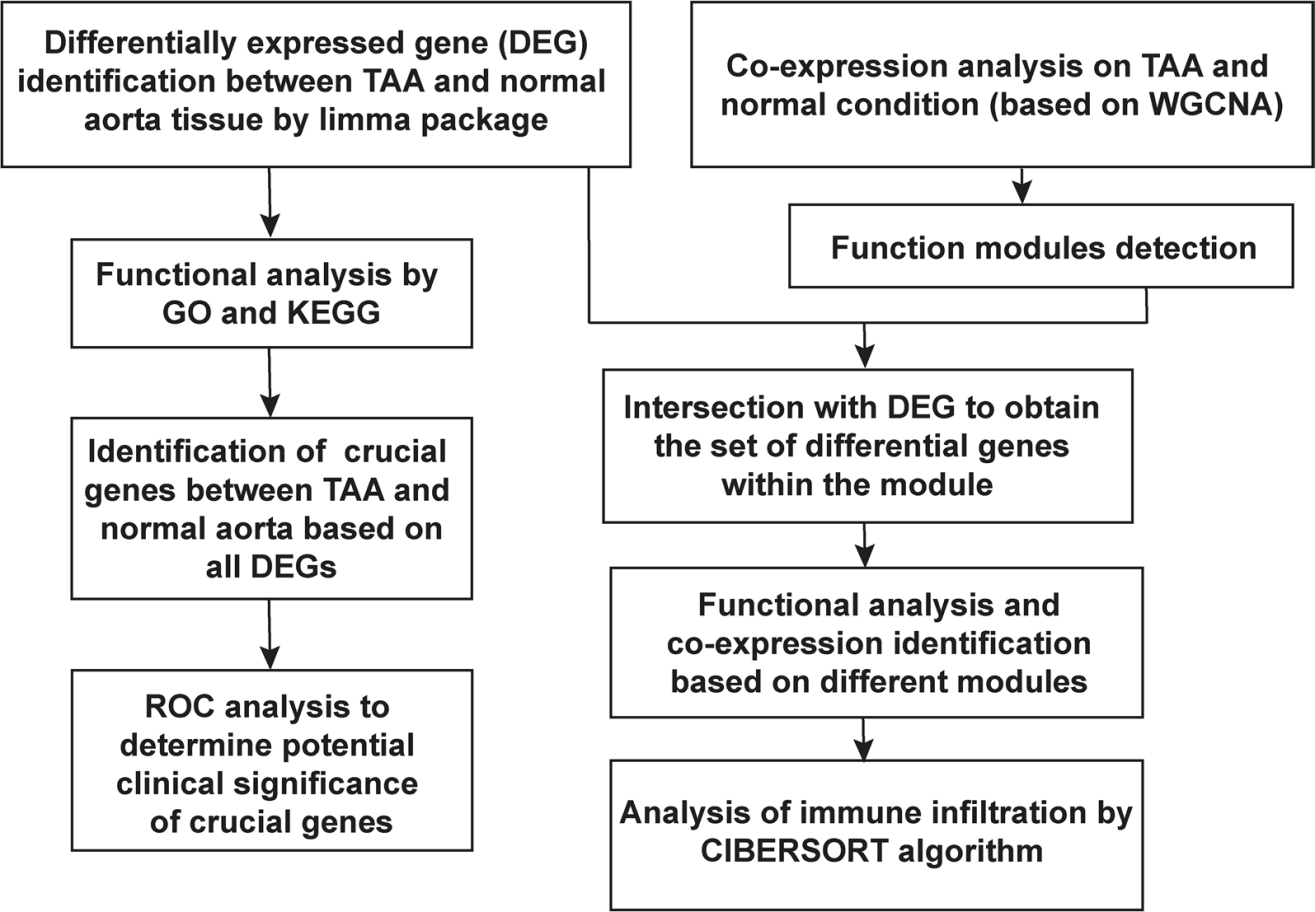
Design and workflow of the whole study

The transcriptome data of TAA patients were obtained from a public database (Gene Expression Omnibus [GEO], GSE26155) and retrospectively analyzed (Fig. 1). Tissue biopsies for the RNA microarray assay were obtained from the nondilated (< 40 mm) and dilated (> 45 mm) aortas of patients with a normal tricuspid aortic valve (TAV). After sorting out the samples, 31 normal thoracic aorta and 28 TAA samples were finally included in this study, and the source of the samples was the media and intima of the artery. The gene expression profiles were identified by Affymetrix ST 1.0 exon microarrays.

### DEG acquisition

The original dataset downloaded from GEO underwent background correction and quantile normalization by the “limma” package (version 3.44.3) [16] in R version 4.0.2. The normalization and Log2 transformation of the transcripts were then performed for the expression profiles with the same package. False discovery rate (FDR) < 0.05 and absolute value of the Log_2_ fold change (Log_2_FC) > 0.5 or > 0.8 were considered statistically significant in different circumstances.

### Functional and pathway enrichment analyses and principal component analysis (PCA)

As was reported previously, Gene Ontology (GO) annotation contains three subontologies—biological process (BP), cellular component (CC), and molecular function (MF), and can identify the biological properties of genes and gene sets for all organisms [17]. Kyoto Encyclopedia of Genes and Genomes (KEGG) is another curated database of well-established biological pathways of gene functions and molecular processes. All the DEGs and the genes of interest identified through the WGCNA were respectively examined for enrichment in these curated pathways and ontologies (R-project version 4.0.2, BiocManager R package version 1.30.10, and clusterProfiler R package version 3.8.1) to identify the possibly related functions and pathways that may mediate the occurrence of TAA. An adjusted *p* < 0.05 was set as the cut-off criterion and Fisher's exact approach was performed to test the gene sets for enrichment. PCA, which can reduce the dimensionality of data and identify the pivotal elements, is performed by a linear transform that extracts the essential elements in the data using a correlation matrix as described in previous studies [18]. PCA was conducted on the normalized expression profiles of all the genes set as well as the DEGs subset to identify differences in the transcriptome patterns during TAA and in the normal aorta.

### Coexpression network construction by WGCNA

A WGCNA network was generated to identify global gene expression profiles as well as coexpressed genes. The soft power threshold β was used to transform the correlation matrix into a scale-free adjacency matrix, and it was determined by the “pickSoftThreshold” function (WGCNA R package [19], version 1.69), and the screening criterion of β was an R-square of the scale-free fit greater than 0.9. Then, we created the weighted adjacency matrix with the formula a_mn_ =|c_mn_|^β^ (where a_mn_ is the adjacency between gene m and gene n, c_mn_ is Pearson’s correlation, and β is the soft power threshold). The topological overlap matrix (TOM) was derived from the resulting adjacency matrix and was used to cluster the modules using the “blockwiseModules” function and the dynamic tree cut algorithm with a height of 0.25, a deep split level of 2, a reassign threshold of 0.2, and a minimum module size of 50. The correlations between the modules and TAA condition were calculated as previously reported [20]. Briefly, the expression profiles of each module were summarized by the module eigengene (ME) and the correlation between the module and clinical status was calculated. The (weighted) Pearson correlation and Student asymptotic *p*-value were calculated by the relative functions in “WGCNA” R package (version 1.69).

### Hub gene identification and receiver operating characteristic (ROC) analysis

Hub genes were identified as the genes with the highest intramodular connectivity, which was determined by topological analysis methods including degree, edge percolated component, maximum neighborhood component and other approaches. In our study, we used the online tool STRING (Search Tool for the Retrieval of Interacting Genes/Proteins, version 11.0) [21], which is designed for predicting protein–protein interactions (PPI) based on the evidence of experiments and database information, to construct a PPI network of all the DEGs as well as DEGs in the WGCNA modules. Genes with a confidence ≥ 0.7 were chosen to build a network model. Then, these PPI networks were imputed into Cytoscape v3.7.1 [22]. The maximal clique centrality (MCC) algorithm, which was reported to be the most effective method of finding hub nodes [23], was used to identify the hub nodes in the network. The MCC of each node in the PPI network was calculated by the plugin “CytoHubba” [23]. The genes with the top 10 MCC values were considered as hub genes and visualized together with their connected nodes in this study. Finally, ROC analysis was performed with the top ten genes in the cluster and the DEGs by the “pROC” package (version 1.16.2) and “ggplot2” package (version 3.1.0) in R-project (version 4.0.2).

### Evaluation of aneurysm-infiltrating immune cells

As Newman et al. previously reported [24], CIBERSORT is a gene expression-based deconvolution algorithm that can accurately quantify the relative levels of distinct immune cell types within a complex gene expression mixture through a set of barcode gene expression values (a “signature matrix” of 547 genes). In this study, we first performed the “normalizeBetweenArrays” function with the method of “quantile” in the “limma” R package (version 3.36.5) to obtain the normalized gene expression data, which was then used to infer the relative proportions of 22 types of infiltrating immune cells. The gene expression datasets were prepared using standard platform annotation files and data uploaded to the CIBERSORT web portal (http://cibersort.stanford.edu/), with the algorithm run using the default signature matrix at 5000 permutations. To ensure the confidence of the results, CIRBERSORT uses Monte Carlo sampling to derive the *P*-value of the deconvolution for each sample, and we also deployed a filter to remove samples with *P*-values less than 0.05 from the data to ensure accurate prediction of the sample. After data processing, there were 27 normal and 26 dilated aorta samples that were included in the follow-up analysis.

### TAA Animal model establishment

See Additional file 1.

### Validation of key genes by quantitative real time–polymerase chain reaction

See Additional file 1.

### Histochemical staining

See Additional file 1.

### Statistical analysis

The differences in gene expression and immune cell infiltration were tested by the Wilcoxon rank-sum test, and Pearson correlation coefficients were calculated to reveal the correlation of any two immune infiltrating cells. The analysis of DEGs was conducted by the “limma” package, and heatmaps were constructed by the “pheatmap” package (version 1.0.12) in R language. Except for special instructions, two-sided *p*-values or FDR less than 0.05 were considered statistically significant. All of the statistical analyses were carried out using SPSS software (version 24.0, IBM SPSS statistics) and R language (R-project.org, version 4.0.2) with packages available through the Bioconductor project (www.bioconductor.org).

## Results

### DEGs and related pathways

**Fig. 2:**
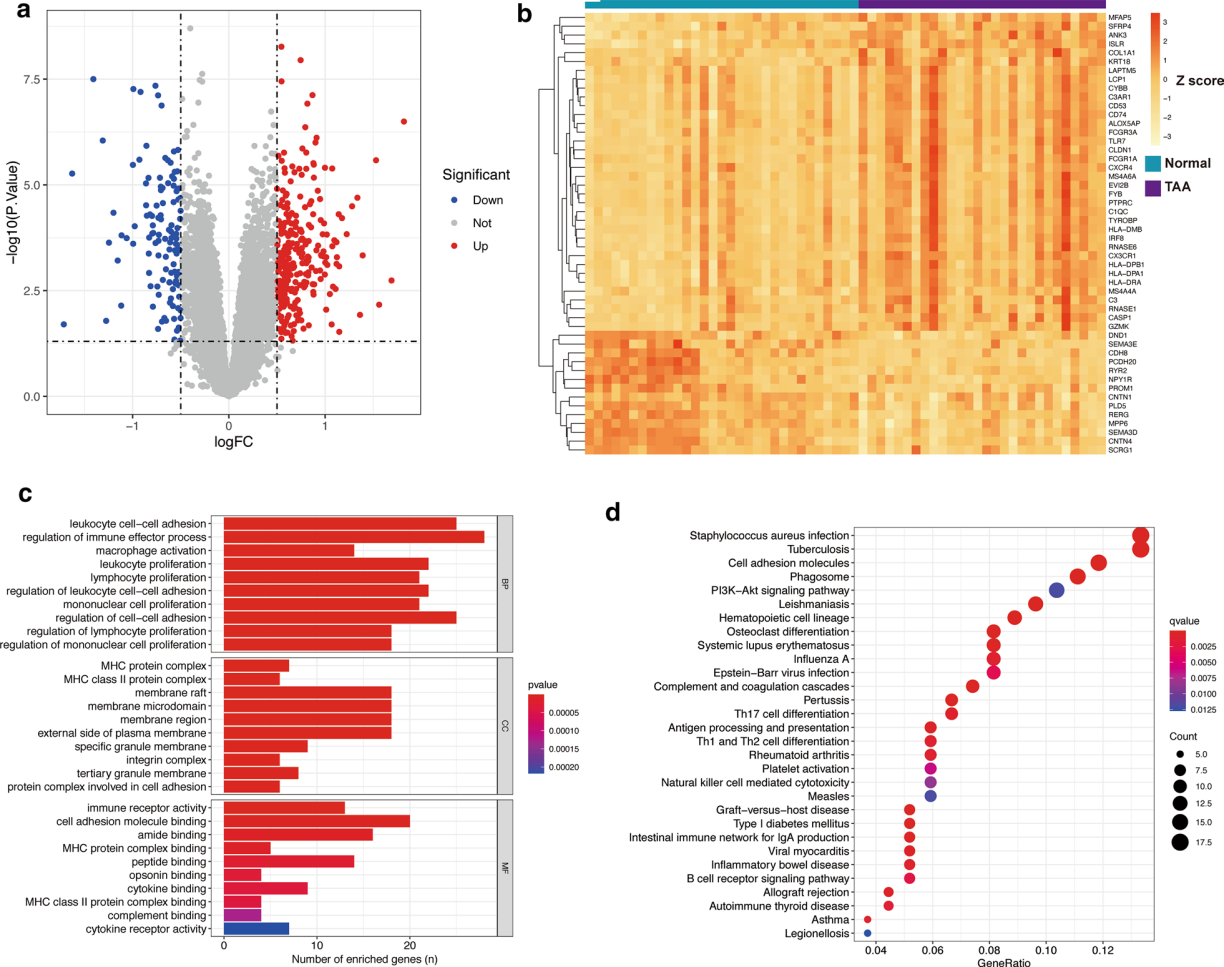
Identification and analysis of differentially expressed genes (DEGs). **a** Volcano plot of all the genes. The dendrogram represents the cluster analysis of the 50 genes. **b** Heatmap plotted by the z-scores of the 50 most significantly differential expressed genes. **c** Gene set enrichment analysis of Gene Ontology (GO) among all the DEGs. BP: biological process; CC: cellular component; MF: molecular function. **d** Gene set enrichment analysis of Kyoto Encyclopedia of Genes and Genomes (KEGG) among all the DEGs. Gene ratio: the ratio of the enriched genes to the total number of genes in the relative pathway in the database

To obtain more comprehensive information, we set the Log_2_FC threshold as the absolute value greater than 0.5. In this circumstance, 85 genes were downregulated and 171 genes were upregulated in the TAA group compared to the normal group (Table 1, Fig. 2a, b, Additional file 2: Table S1). To further investigate the biological behaviors of these DEGs, we performed enrichment analysis on GO (Fig. 2c) and KEGG (Fig. 2d) gene sets (GO and KEGG ids and other detailed information are provided in the supplementary materials, Additional file 3: Table S2). According to the enrichment analysis, regulation of the immune effector process, cell–cell adhesion, immune cell proliferation, and immune receptor activity were most significantly enriched, suggesting that the immune process might play an essential role in TAA formation and development. Accordingly, enrichment analysis of KEGG pathways implied that Th17, Th1 and Th2 cell differentiation, natural killer (NK) cell-mediated cytotoxicity, and the B cell receptor signaling pathway might participate in the disease process. However, it also suggested that some bacterial or viral infections, such as Staphylococcus aureus, tuberculosis, influenza A, and Epstein-Barr virus, could be accompanied by the formation or progression of TAA.

**Table 1:** The top 30 most differentially expressed genes between normal and dilated thoracic aortic aneurysms (TAAs)

Gene symbol	Log_2_(FC)^a^	FDR	Gene symbol	Log_2_(FC)^a^	FDR^¶^
CX3CR1	1.818	< 0.001^***^	SEMA3D	− 1.628	0.001^**^
HLA-DPA1	1.529	< 0.001^***^	PCDH20	− 1.408	< 0.001^***^
C3	1.390	0.013^**^	CDH8	− 1.310	< 0.001^***^
CD74	1.335	0.003^**^	CNTN1	− 1.246	0.009^**^
HLA-DRA	1.280	0.003^**^	DND1	− 1.199	0.004^**^
CD53	1.224	0.007^**^	PROM1	− 1.155	0.015^*^
FCGR3A	1.175	0.005^**^	CNTN4	− 1.115	0.007^**^
MFAP5	1.146	0.018^*^	RYR2	− 1.062	0.008^**^
C3AR1	1.141	0.004^**^	SEMA3E	− 0.997	0.001^**^
MS4A4A	1.122	0.014^*^			
LAPTM5	1.118	0.017^*^			
EVI2B	1.118	0.009^**^			
HLA-DPB1	1.095	0.009^**^			
CYBB	1.075	0.010^*^			
SFRP4	1.074	0.001^**^			
LCP1	1.022	0.017^*^			
C1QC	1.017	0.007^**^			
ALOX5AP	1.011	0.013^*^			
PTPRC	1.001	0.016^*^			
TLR7	0.998	0.001^**^			
CXCR4	0.997	0.023^*^			

Log_2_FC: Log_2_ fold change; FDR: false discovery rate

^¶^FDR was calculated by *p* value from Wilcoxon test

^*^marked significant differences. *FDR < 0.05, **FDR < 0.01, ***FDR < 0.001

^a^Log_2_FC = Log_2_(mean expression of TAA group/ mean expression of control group)

### Construction of the coexpression network of TAA and normal conditions

**Fig. 3:**
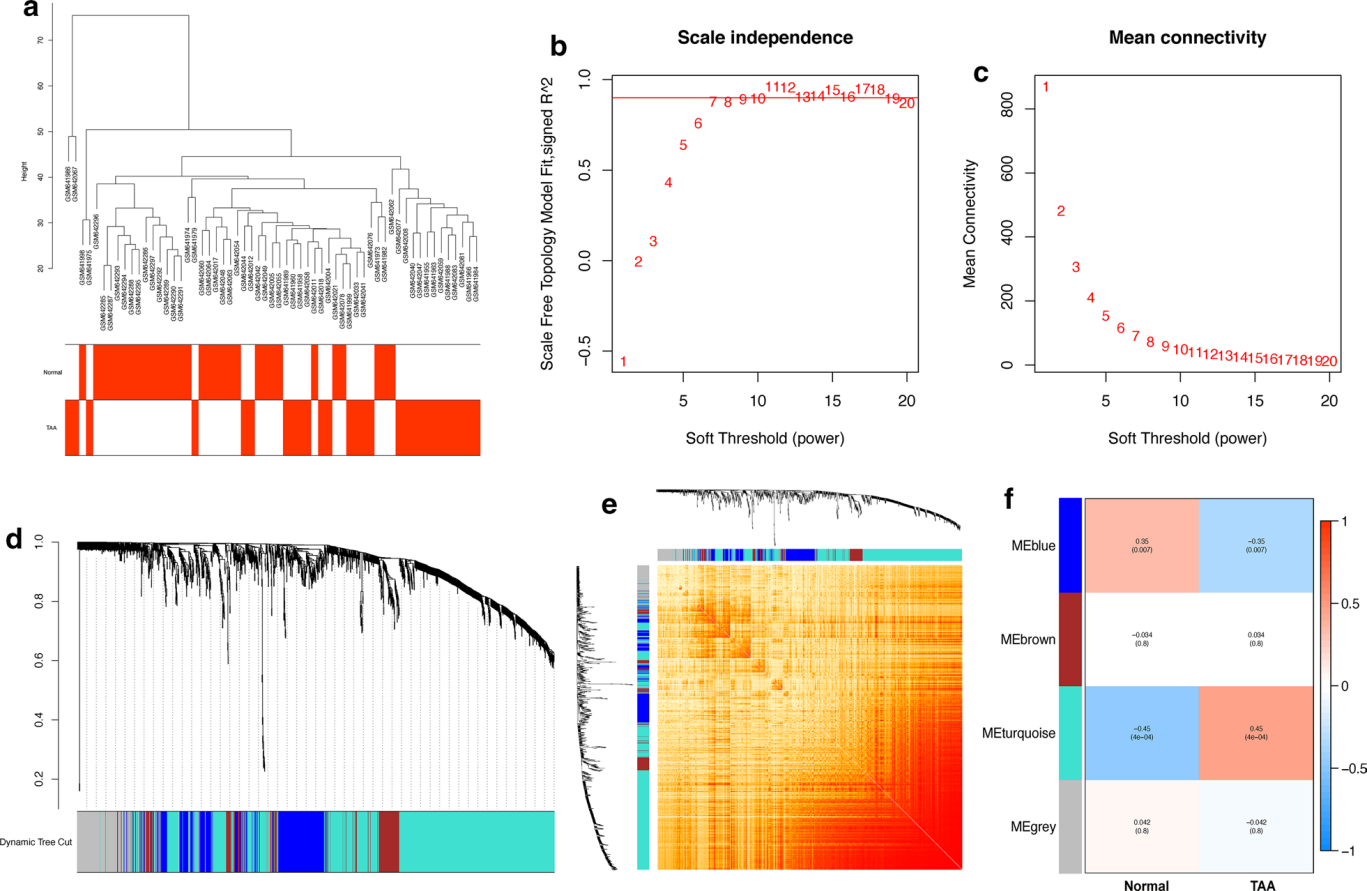
WGCNA on TAA and normal conditions. **a** Detection of outliers with sample clustering. All samples are located in the clusters and pass the cutoff thresholds. **b**, **c** Soft threshold power analysis implemented to obtain the scale-free fit index of the network topology, with a soft threshold power β of 7. **d** Hierarchical cluster analysis detection of the coexpression clusters determined by WGCNA. **e** Heatmap of the topological overlap matrix (TOM) of genes selected for WGCNA. The red color represents a higher overlap. **f** Diagram of correlation between different modules and disease status. The turquoise module is significantly positively correlated with TAA, and the blue module is significantly negatively correlated with TAA. The red color represents the positive coefficient factor and the blue color indicates the negative coefficient factor

To further identify the expression patterns of TAA compared to normal thoracic aorta, we conducted PCA on the overall genes and the DEGs. According to 2D-PCA among all the genes, PC1 and PC2 accounted for 24.4% and 9.6% of the variation in the gene expression values (Additional file 1: Figure S1A, Additional file 4: Table S3). In the 2D-PCA of DEGs, the variation proportions of PC1 and PC2 were 55.9% and 10.2%, respectively Additional file 1: Figure S1B, Additional file 4: Table S3. The 2D-PCA plot showed that variation between the TAA and normal groups indeed existed (Additional file 1: Figure S1C, D). Therefore, we conducted WGCNA on TAAs and normal aortas to explore the coexpression network. The sample clustering dendrograms of the TAA and normal conditions are shown in Fig. 3a and all samples are located in the clusters and pass the cutoff thresholds. The soft threshold value β = 7 was selected for transformation of the correlation matrix into a scale-free adjacency matrix (Fig. 3b, c). The gene modules were detected based on the TOM, and four modules were finally detected (Fig. 3d, e). To further detect the correlation of different modules with the TAA condition, we calculated the correlation factors of each module. It suggested that the blue module had the most significant negative correlation with TAA (correlation coefficient = − 0.35, *p* = 0.007, containing 340 genes, Additional file 5 Table S4), while the turquoise module, which contains 992 genes, had the most significant positive correlation with TAA (correlation coefficient = 0.45, *p* < 0.001) The correlation with the normal condition of each module was the opposite of that with the TAA. The modules obtained by WGCNA were verified with the results of cluster analysis of differential genes.

### Identification of the functional pathways related to the crucial modules

**Fig. 4:**
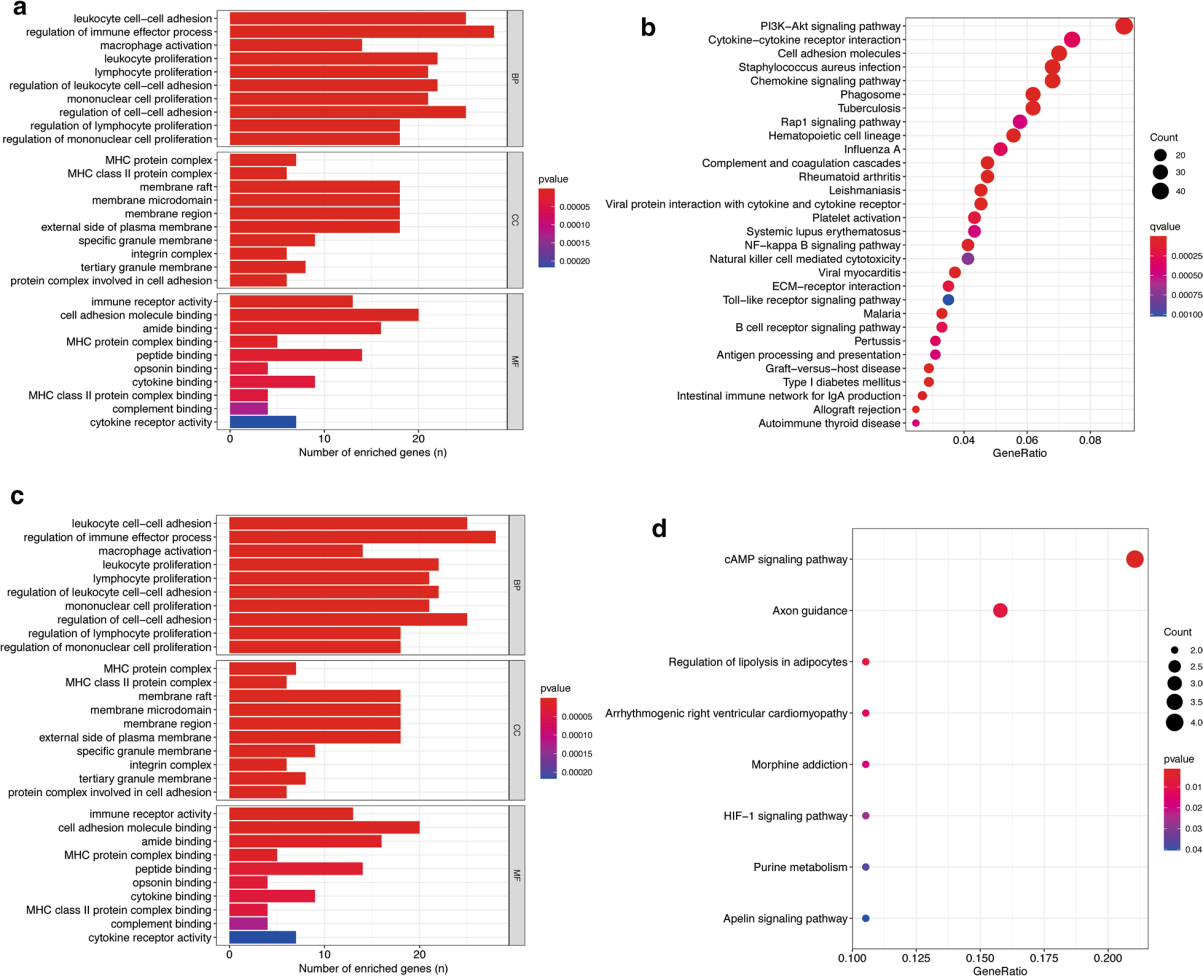
Gene set enrichment analysis of GO and KEGG ontologies and pathways among DEGs in blue and turquoise modules. **a,** **b **GO and KEGG of DEGs in the turquoise module. **c**, **d** GO and KEGG of DEGs in the blue module

To further explore the physiological or pathological pathways related to TAA, the DEGs between TAA and normal tissues were intersected with the genes in turquoise and blue modules respectively. We performed gene set enrichment analysis of GO and KEGG ontologies and pathways on these genes. The genes in the turquoise module, which were significantly associated with TAA, were enriched in several pathways, including leukocyte, lymphocyte, and mononuclear cell proliferation, the cytokine-cytokine receptor interaction, and the B cell receptor signaling pathway (Fig. 4a, b; detailed information is provided in Additional file 6: Table S5). On the other hand, a similar analysis conducted on the DEGs in the blue module suggested that the negative regulation of cell adhesion and chemotaxis, the response to redox state, the cAMP signaling pathway and several other functional pathways were significantly related to normal conditions (Fig. 4c, d; detailed information is provided in Additional file 7: Table S6).

### Identification of the hub genes of the DEGs and functional modules

**Fig. 5:**
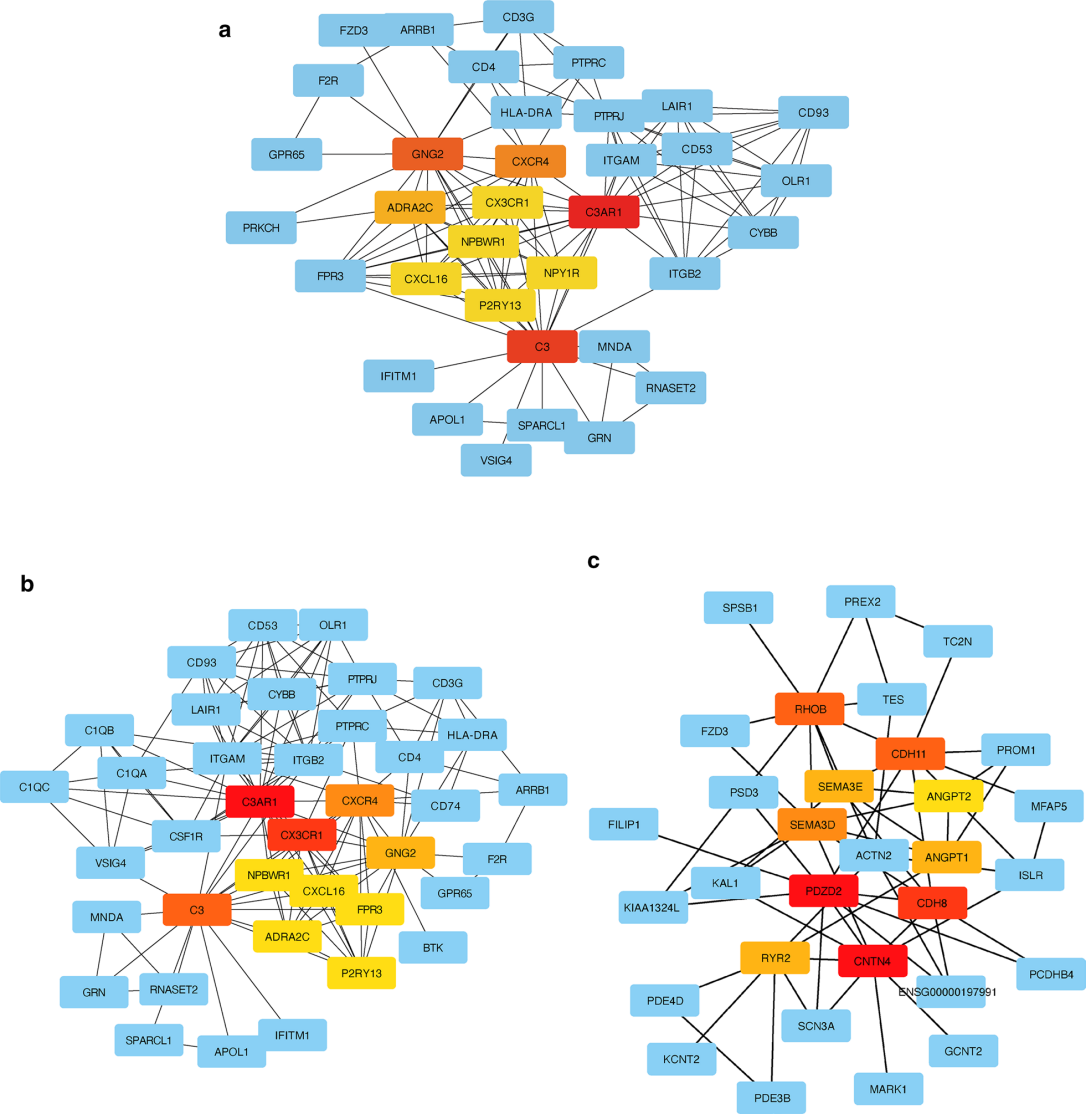
Hub genes identification by the CytoHubba algorithm. **a** The top 10 hub genes ranked by the MCC algorithm and their neighbors among all the DEGs. **b** The top 10 hub genes ranked by the MCC algorithm and their neighbors in the turquoise module. **c** The top 10 hub genes ranked by the MCC algorithm and their neighbors in the blue module

Genes that play a vital role in biological processes are defined as hub genes. In related pathways, the regulation of other genes is often affected by these genes. The DEGs in the blue and turquoise modules were inputted into the STRING database to construct PPI networks (Additional file 1: Figure S2). We implemented the MCC algorithm by CytoHubba and the genes with the top 10 MCC values were considered as hub genes and visualized together with their connected nodes. Interestingly, the hub genes of the DEGs and turquoise module largely overlapped (Fig. 5a, b), and *C3AR1*, *CX3CR1*, and *C3* were the top three genes ranked by the MCC algorithm in CytoHubba. However, the topological network of the hub genes of the blue module was different from the others (Fig. 5c). The top genes ranked by the MCC algorithm in the blue module consist of *CNTN4*, *PDZD2*, and *CDH8*.

### Different immune infiltrative patterns between TAA and normal conditions

**Fig. 6:**
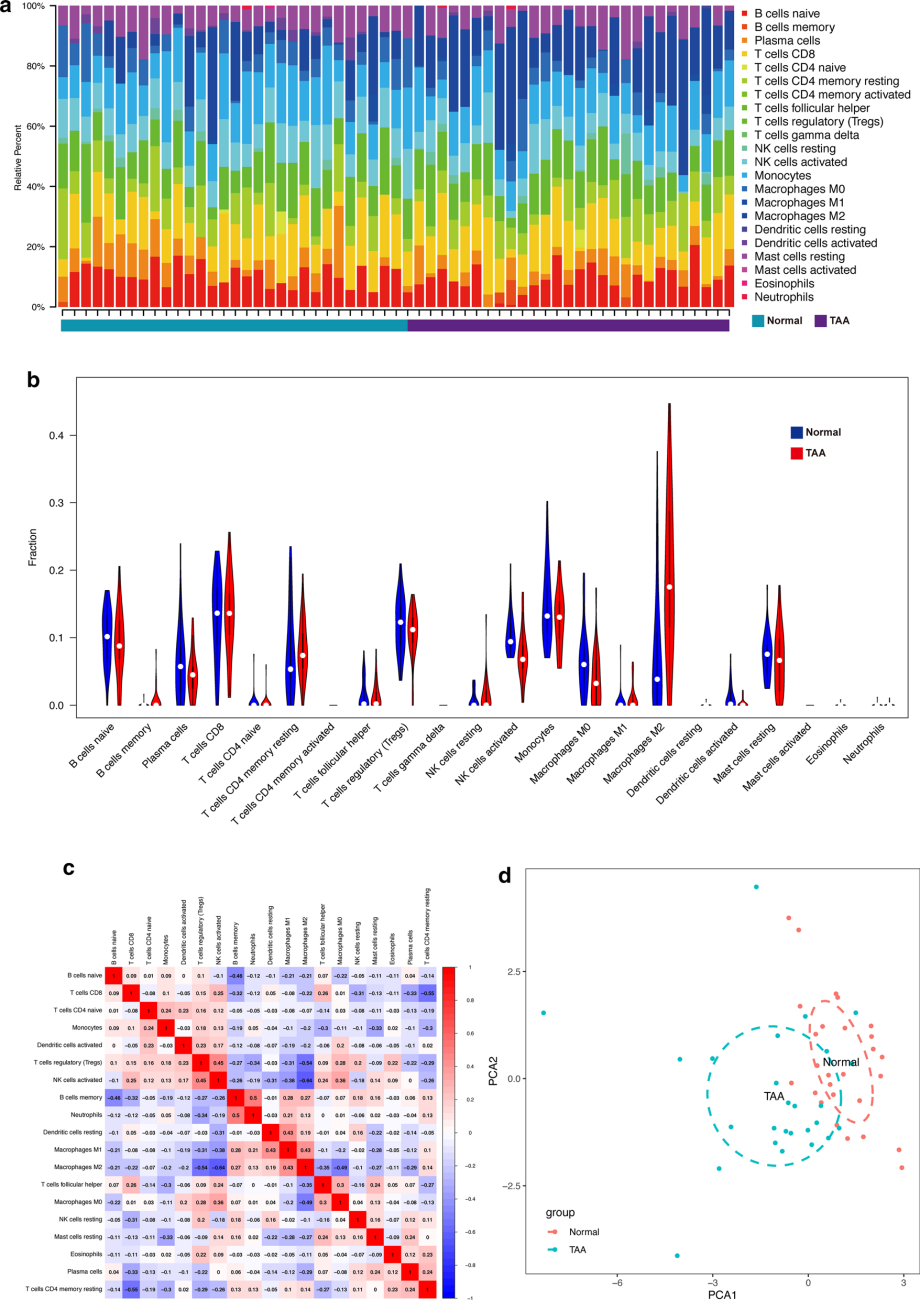
Immune infiltrative patterns in TAA and normal conditions. **a** Histogram of 22 types of immune cells in each TAA and normal tissue. **b** Violin chart showing the differences in infiltrating immune cells between the two groups. The blue color represents the normal condition and the red color represents the TAA. **c** Correlation heatmap of all the immune cells. The numbers in the small square represent Pearson’s correlation coefficient between the two immune cells on the horizontal and vertical coordinates. **d** Principal component analysis (PCA) suggests a difference between TAA and normal thoracic aorta in the composition of infiltrating immune cells, which it might not be significant

According to the gene set enrichment analysis of GO and KEGG ontologies and pathways, several pathways related to immune processes were identified. Therefore, we used CIBERSORT to evaluate the patterns of immune infiltration in TAA and under normal conditions. The relative proportions of 22 immune cells were detected in the TAA and normal thoracic aorta samples (Fig. 6a). The proportion of M0 macrophages was higher under normal conditions, while those of M1 and M2 macrophages exhibited the opposite trend (though both groups had a trace content of M1 macrophages). In addition, the proportion of activated NK cells was dramatically higher under normal conditions (Fig. 6b). Accordingly, activated NK cells were negatively correlated with M2 macrophages (Fig. 6c). To summarize, the 2D-PCA plot suggested that the patterns of immune infiltration in TAA and under normal conditions were different (Fig. 6d).

### Verification of crucial genes in the murine TAA model and ROC analysis

**Fig. 7:**
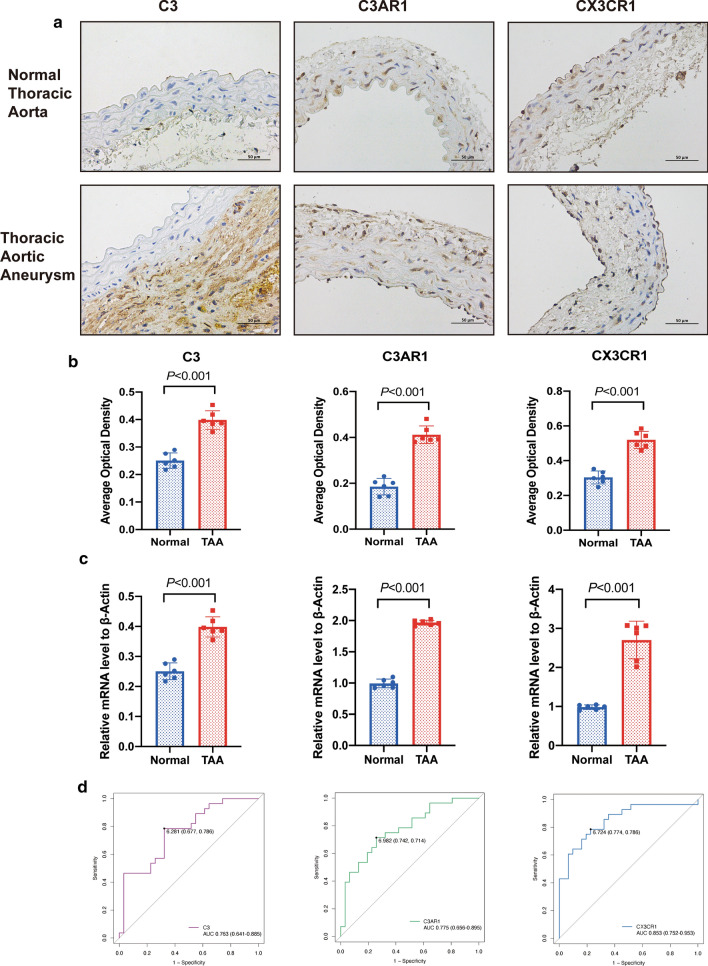
Verification of crucial hub genes in the murine model. **a**, **b** Immunohistochemical staining of *C3, C3AR1, CX3CR1* and the representative figures. **c** RT-qPCR of the top three hub genes in animal model. **d** ROC analysis of the three hub genes

We examined the protein expression by immunohistochemical staining (Fig. 7a, b) and the mRNA amount by RT-qPCR (Fig. 7c). The top three hub genes were significantly highly expressed in TAA compared to normal aorta, which was in line with the bioinformatic analysis and further validated the results. Finally, ROC analysis was conducted to explore potential biomarkers. Since the hub genes in the turquoise module overlapped with the DEGs to a large extent, we selected 10 hub genes from the DEGs for ROC analysis (Fig. 7d and Additional file 1: Figure S3). All the areas under the curve (AUCs) were greater than 0.7, and *ADRA2C*, *CX3CR1*, and *NPBWR1* had the highest AUCs (AUCs = 0.862, 0.853 and 0.810, respectively).

## Discussion

To identify the essential modules that were closely related to TAA, we performed WGCNA on TAA and normal samples together. WGCNA suggested that two modules were significantly related to the disease condition. Functional enrichment analysis of intersecting genes between the modules and DEGs indicated that immune processes might play an essential role in TAA formation and progression. Further verification in murine TAA model and ROC analysis indicated that the top ten DEGs have potential diagnostic utility and are potential biomarkers. Furthermore, we detected the hub genes and performed immune infiltration analysis, which further suggested that *C3AR1, CX3CR1, and C3,* along with activated NK cells and M2 macrophages, could be the driving factors of the disease or potential therapeutic targets.

In this study, we intersected genes between DEGs and different modules to identify genes in these modules that differentially expressed between TAA and normal tissue. There were 992 genes in the turquoise module, and most of the DEGs were included (204 of 256 DEGs). Moreover, the turquoise module was significantly associated with TAA, indicating the central role of these genes in the occurrence and development of TAA. Recently, a large number of research results have reached a consensus that inflammation is an integral part of the pathophysiological process in aneurysmal disease and aortic dissection, and leukocytic infiltration is closely associated with medial degeneration and VSMC death in aneurysmal aortas [25–27]. According to our data, C-X3-C Motif Chemokine Receptor 1 (*CX3CR1*) was one of the hub genes in the turquoise module, and its expression was significantly higher in TAA than in normal aorta, with a Log_2_FC of 1.818 (FDR < 0.001). *CX3CR1* is the receptor of fractalkine (CX3CL1) and is involved in the adhesion and migration of leukocytes [28, 29]. In human coronary atherosclerosis, fractalkine is expressed at a high level by a subset of inflammatory cells. These inflammatory cells in the intima were directly correlated with the number of CXCR1^+^ cells, most of which were VSMCs [30]. In addition, angiotensin II (Ang II) dysregulation was determined in many cardiovascular diseases, and it could upregulate CX3CR1 expression in VSMCs via the NADPH oxidase/ROS/p38 MAPK pathway [31]. Interestingly, CX3CL1^+^ and CX3CR1^+^ cells are also present in AAAs, indicating their contributions to the recruitment of inflammatory cells [32]. Consistent with these results, the functional enrichment analysis of the DEGs in our study suggested that leukocyte adhesion might play a critical role in TAA.

Another two hub genes detected by WGCNA, C3 and its receptor C3AR1, are essential molecules in the complement system and are closely related to innate immune activities. C3 is cleaved by C3 convertase, at which all three complement activation pathways converge [33]. Several studies have reported that the activation of the complement system participates in human AAA and thoracic aortic dissection (TAD) [34–36]. Furthermore, the level of C3 degradation products was elevated in human AAA intraluminal thrombi but to a lesser extent in the AAA wall, likely indicating the increased proteolysis of C3 by proteases present in AAA [34]. Similarly, C3a, which is the degradation product of C3, was significantly upregulated in TAD patients and murine models. C3AR was correspondingly elevated in the smooth muscle cells of dissected aortas. Knockout of C3aR decreased the matrix metalloproteinase 2 (MMP2) expression in mice induced by β-aminopropionitrile monofumarate (BAPN) and notably inhibited the formation and rupture of TAD [36]. According to the STRING database, C3AR has a strong interaction with CD93 (with a combined score of 0.96), which was slightly more highly expressed in TAA, with a Log_2_FC of 0.756 (FDR < 0.001). Again, CD93 was associated with the innate immune system and immune response lectin-induced complement pathway. It was previously found that murine CD93 contributes to the removal of apoptotic cells in vivo [37]. Interestingly, Strawbridge et al. demonstrated the function of soluble CD93 (sCD93) in glucometabolic regulation, and lower sCD93 levels might be related to type 2 diabetes patients who have a higher cardiovascular disease risk [38]. However, no research has focused on the mechanism of action of CD93 molecules in aneurysmal diseases.

In this study, we confirmed the relevant pathways of monocyte/macrophage proliferation through functional enrichment analysis, and the result was further verified by CIBERSORT. Previous results have shown that macrophage accumulation in the aneurysmal aortic wall is involved in the production of chemokines and cytokines in response to tissue injury [39–41]. Recently, several macrophage phenotypes were determined, and the dichotomy to divide macrophages into M1 and M2 macrophages has been widely applied [42–44]. Our data suggested that the proportion of M2 macrophages was much higher in TAA than in normal tissues, which was consistent with the observation of the former study [45]. More recently, single-cell transcriptome analysis revealed a heterogeneous distribution of monocytes/macrophages derived from human ascending TAAs and normal tissues [46]. Due to the chronicity of the disease, however, macrophage infiltration and polarization might evolve during several steps in the development and progression of aneurysms, and further and more in-depth studies are needed to identify the macrophage phenotype spectrum in aneurysmal diseases.

The blue module, on the other hand, contained most of the downregulated DEGs. The functional enrichment analysis showed that negative regulation and chemotaxis might provide a protective effect. For instance, cadherin 8 (*CDH8*) was markedly downregulated in TAA (Log2FC =  − 1.310, FDR < 0.001) and was one of the hub genes in the blue module. CDH8 protein is an integral membrane protein that mediates calcium-dependent cell–cell adhesion [47]. Additionally, *CDH11*, which is the paralog gene of *CDH8*, was slightly upregulated in TAA (Log_2_FC = 0.697, FDR = 0.004). However, the functions and mechanisms of both CDH8 and CDH11 in aneurysmal diseases have not been clarified. Our work sheds light on the molecular basis of the cadherin family in TAA. Future investigations on this basis will likely illuminate novel potential treatment targets of aneurysmal disease. To our surprise, the proportion of activated NK cells was notably lower in the TAA group. These results contrast with the findings of Foreste et al. [48] and Hinterseher et al. [49], which might be explained by the different sources of NK cells (peripheral blood and AAA tissue NK cells) and the differences in the methods and markers used to identify NK cells. Our study suggests that the formation or development of TAA may involve cell and molecular mechanisms different from those in AAA.

There are several limitations to our study. First, it is difficult to evaluate the reliability of the original samples due to the nature of secondary analysis. The relatively small sample size might introduce some bias in the results of comparisons, which included DEGs, WGCNA, and infiltrating immune cell estimation. Additionally, there were observed differences in gene expression profiles between TAA and normal tissue, thus, the combination of TAA and normal tissue may lead to a decrease in connectivity within the module and generation of larger modules. To narrow down the range of candidate hub genes, we performed a follow-up analysis only among the DEGs in the different modules. Although we tried to avoid this limitation by defining a smaller threshold to include more DEGs, some information contained in genes that are not significantly differentially expressed may be lost. We noted that there were 992 genes in the turquoise module, including most of the DEGs (204/256), suggesting that there may be some bias in gene allocation by DEGs alone, and a better allocation method needs to be identified in future studies. Additionally, the specimens were from the intima and media, which may also introduce some bias into the study. Finally, all the analyses were based on the bioinformatics algorithm, and relevant clinical and experimental samples are necessary to verify the results in a future study. In summary, further research is needed to provide more direct evidence of the molecular mechanisms and immune infiltration of TAA.

## Conclusion

In this study, we introduced an efficient method to evaluate the coexpression network of TAA and normal conditions. Several DEGs and two modules with remarkable correlation to disease were identified. Functional pathways of the immune response, including leukocyte cell–cell adhesion, macrophage activation, lymphocyte and other immune cell proliferation, were notably enriched on gene sets of GO and KEGG. Several hub genes related to chemotaxis and cytokines might play a central role in TAA formation and progression. Further analysis identified that macrophages and NK cells could be the driving factors of TAA, and they are worthy of further and more in-depth research.

## Supplementary Information

**Additional file 1: Supplementary Figures and Methods. Figure S1.** Principal Component Analysis (PCA) on the overall genes and the DEGs. Proportions of variance of PCs in PCA among all genes **(A)** and DEGs **(B)**. 2D-PCA plot of all genes **(C)** and DEGs **(D)**. **Figure S2.** Functional protein association network of all DEGs **(A)**, turquoise module **(B)** and blue module **(C). Figure S3.** ROC analysis of the top 10 DEGs. **A-J.** ROC curves for *GNG2, C3AR1, CX3CR1, ADRA2C, C3, CXCL6, NPBWR1, P2RY13, NPY1R, CXCR4*.

**Additional file 2. Table S1.** Differentially expressed genes (DEGs) in TAA compared to normal condition.

**Additional file 3. Table S2.** Enrichment analysis on GO and KEGG gene sets among DEGs.

**Additional file 4. Table S3.** Summary of the 2D-PCA among all genes and DEGs.

**Additional file 5. Table S4.** List of genes in the four modules.

**Additional file 6. Table S5.** Enrichment analysis on GO and KEGG gene sets among DEGs in the turquoise module.

**Additional file 7. Table S6.** Enrichment analysis on GO and KEGG gene sets among DEGs in the blue module.

## Data Availability

The datasets used and/or analyzed during the current study are available from the corresponding author on reasonable request.
