# Supplementary material for: The potential role of chemotaxis and the complement system in the formation and progression of thoracic aortic aneurysms inferred from the weighted gene coexpression network analysis
Source: J Transl Med. 2021 Feb 2;19:49. doi: 10.1186/s12967-021-02716-6 (PMC7852290; doi:10.1186/s12967-021-02716-6)
Supplement: Supplementary file 1 — Additional file 1: Supplementary Figures and Methods. Figure S1. Principal Component Analysis (PCA) on the overall genes and the DEGs. Proportions of variance of PCs in PCA among all genes (A) and DEGs (B). 2D-PCA plot of all genes (C) and DEGs (D). Figure S2. Functional protein association network of all DEGs (A), turquoise module (B) and blue module (C). Figure S3. ROC analysis of the top 10 DEGs. A-J. ROC curves for GNG2, C3AR1, CX3CR1, ADRA2C, C3, CXCL6, NPBWR1, P2RY13, NPY1R, CXCR4. [file 12967_2021_2716_MOESM1_ESM.docx]

**Additional file 1:**

**Title:** The Potential Role of Chemotaxis and Complement System in the Formation and Progression of Thoracic Aortic Aneurysms Inferred from the Weighted Gene Co-Expression Network Analysis.

**Authors:**

Chuxiang Lei B.S.^1^, Dan Yang Ph.D.^2^, Wenlin Chen B.S.^3^, Haoxuan Kan B.S.^1^, Fang Xu B.S.^1^, Hui Zhang M.D.^1^, Wei Wang M.D.^1^, Lei Ji M.D.^1^, Yuehong Zheng M.D.^1,*^.

**Authors Address Information**

1. Department of Vascular Surgery, Peking Union Medical College Hospital, Peking Union Medical College and Chinese Academy of Medical Sciences, No 1. Shuaifuyuan, Dongcheng District, Beijing, China

2. Department of Computational Biology and Bioinformatics, Institute of Medicinal Plant Development, Chinese Academy of Medical Sciences and Peking Union Medical College, Beijing, China

3. Department of Neurosurgery, Peking Union Medical College Hospital, Chinese Academy of Medical Sciences and Peking Union Medical College, Beijing, China.

* Corresponding author

Correspondence

Yuehong Zheng

TEL: +8613811015811

Email: [yuehongzheng@yahoo.com](mailto:yuehongzheng@yahoo.com)

**Additional file 1: Figures**

**
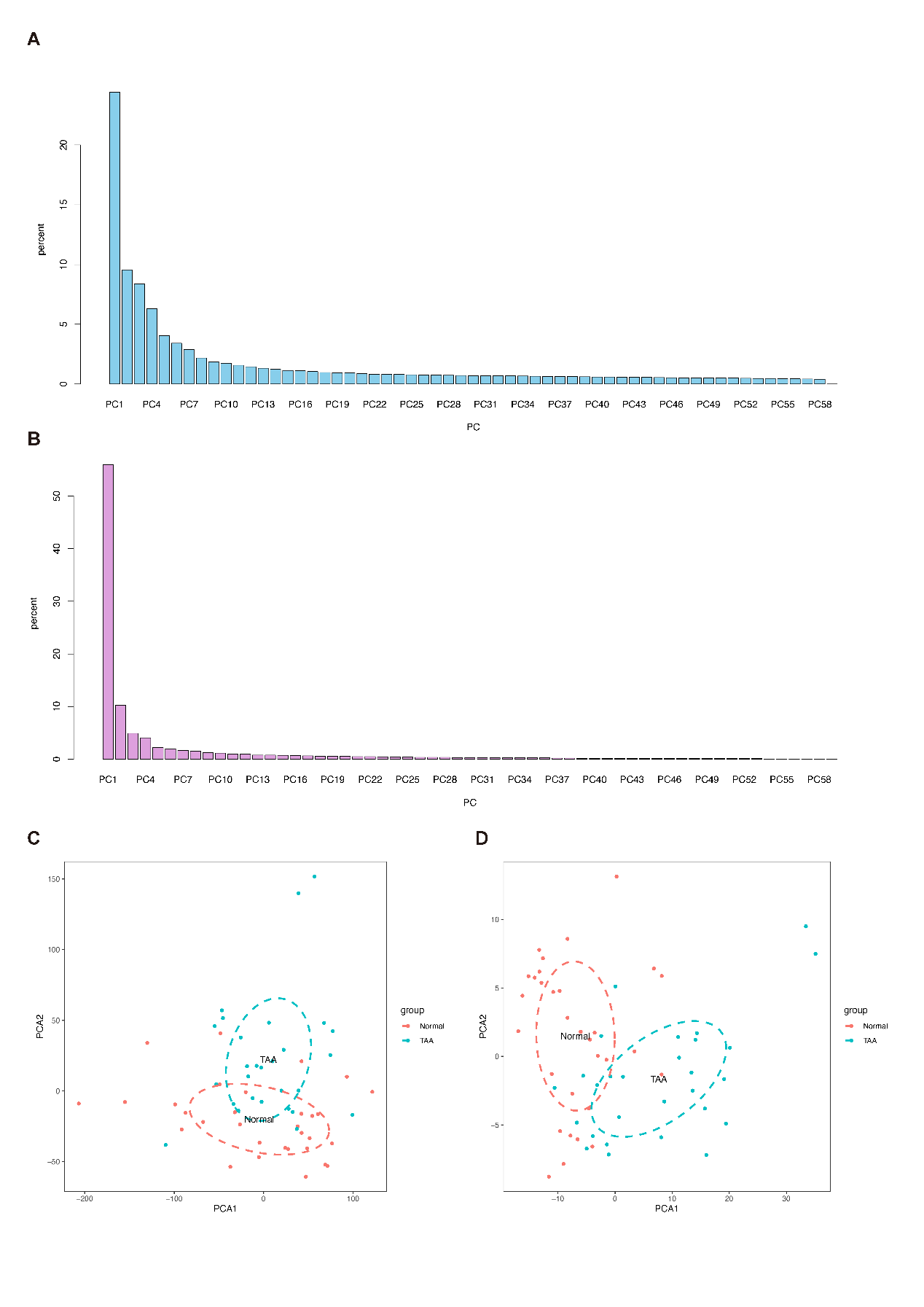
**

**Additional file 1: Figure S1. Principal Component Analysis (PCA) on the overall genes and the DEGs.** Proportions of variance of PCs in PCA among all genes (A) and DEGs (B). 2D-PCA plot of all genes (C) and DEGs (D).


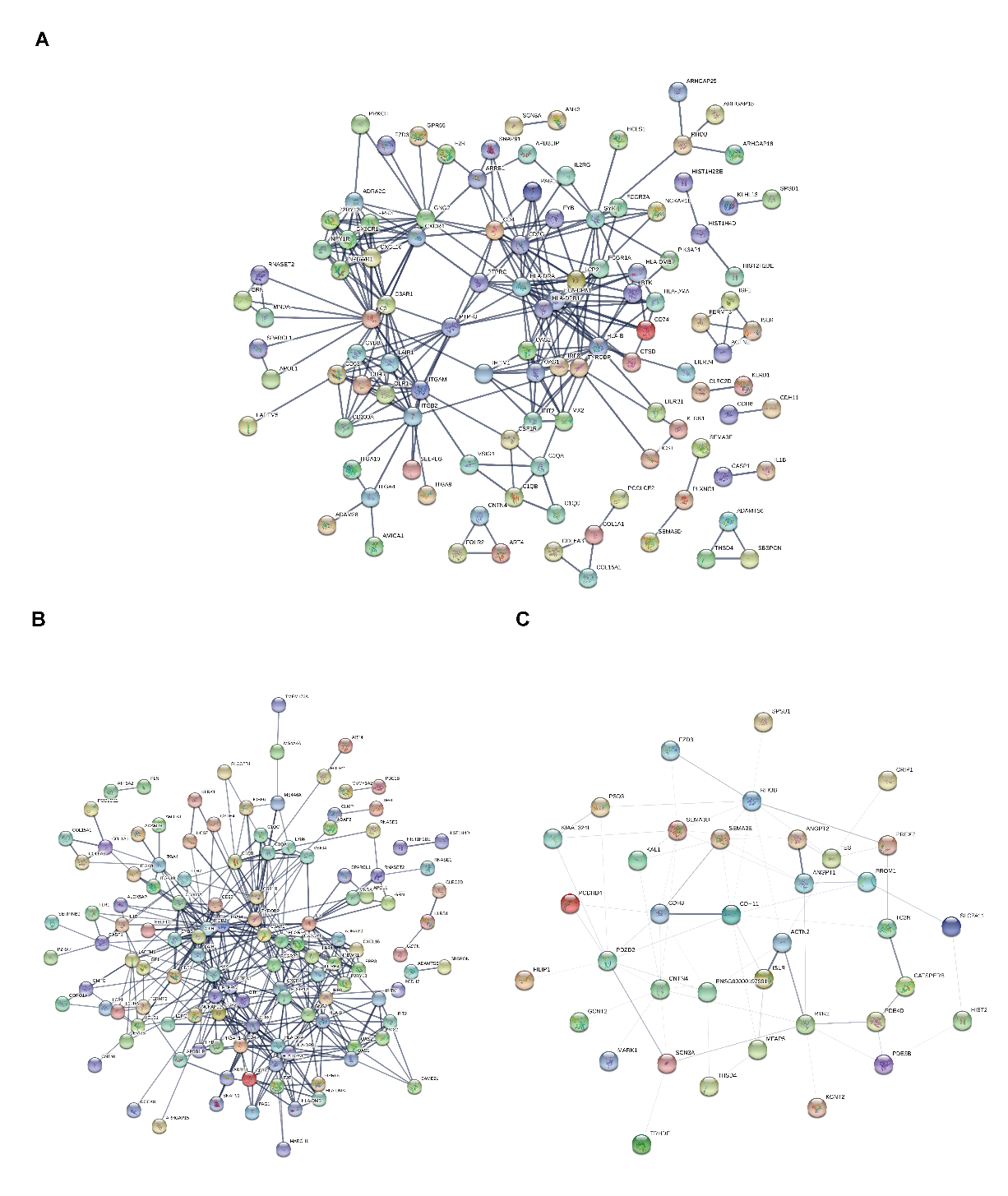


**Additional file 1: Figure S2. Functional protein association network of all DEGs (A), turquoise module (B) and blue module (C).**


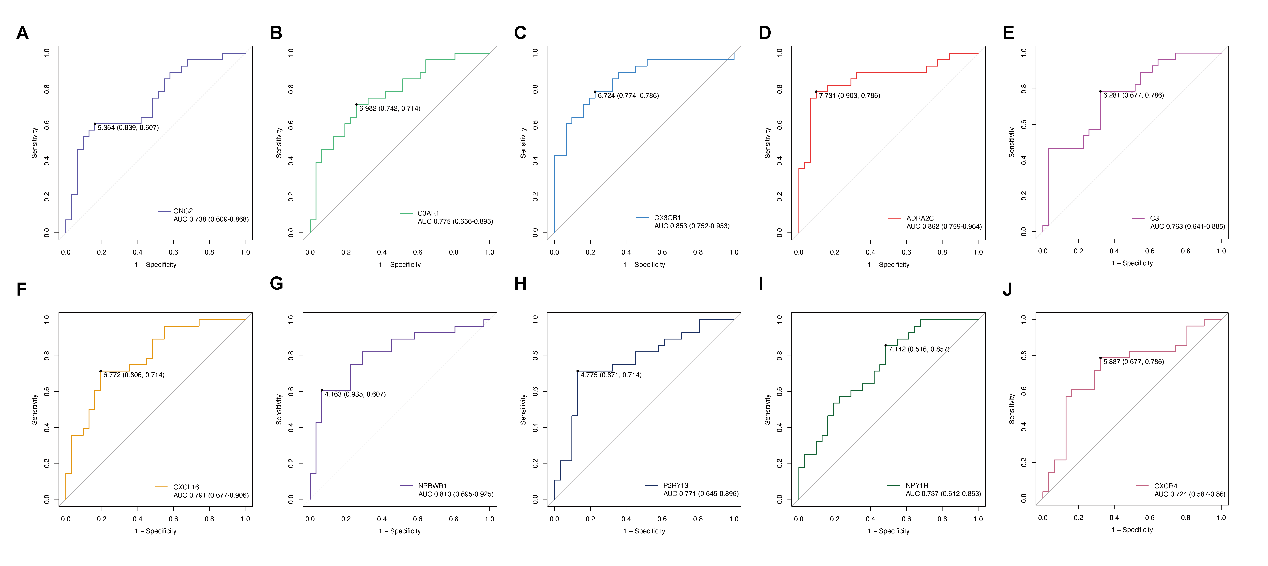


**Additional file 1: Figure S3.** **ROC analysis of the top 10 DEGs. A-J.** ROC curves for *GNG2, C3AR1, CX3CR1, ADRA2C, C3, CXCL6, NPBWR1, P2RY13, NPY1R, CXCR4*.

**Table S1-S6 are attached with separate documents.**

**Additional file 1: Method**

*TAA Animal model establishment.*

Three-week-old male C57BL/6J mice purchased from the Vital River Laboratory Animal Technology Company (Beijing, China) were fed with a normal diet and administered β-aminopropionitrile fumarate (BAPN, #A1314; Sigma-Aldrich, St. Louis, Mo) dissolved in the drinking water at a concentration of 1 g/kg/d for 4 weeks as described previously[1]. At the endpoint of the TAA model, the mice were anesthetized with 1% sodium pentobarbital (40mg/kg). Then, we opened the thoracic cavities of mice, and the blood was drawn from the right ventricle before the integral aortas were harvested.

*Validation of key genes by quantitative real-time polymerase chain reaction*

We performed quantitative real-time polymerase chain reaction (qRT-PCR) of the essential hub genes to further examine the authenticity. Total RNAs were isolated from ground cryopreserved tissues with TRIzol (Invitrogen), and reverse transcription was performed with the FastQuant RT Kit (TIANGEN) according to the manufacturer’s guidelines. qRT-PCR was performed with the CFX Connect™ Real-Time PCR Detection System (Bio-Rad) and reagents from Thermo Scientific (Cat. No. AB1158B). β-Actin was used as control, and the relative expression level was calculated.

*Histochemical staining*

After the integral aortas were harvested, thoracic aortic tissues were perfusion-ﬁxed with 4% neutral buﬀered polyformaldehyde and embedded in paraﬃn. As described previously[2], all sections were cut into 6μm thick slices and were soaked with sodium citrate repair solution three times. Endogenous peroxidase activity was quenched with 3% H_2_O_2_ for 25 min, and sections were blocked with 3% BSA (EPSILON, Cat. No. G5001). Slides were incubated with primary antibodies (anti-C3 1:50; anti-C3AR1 1:50, and anti-CX3CR1 1:100), overnight at 4 °C. The biotin-conjugated secondary antibodies were added for 1 hour at room temperature. Visualization was aided by DAB (EPSILON, Cat. No. G1211) and each slide was photographed under a Nikon DS-U3 microscope at 200× and 400× magniﬁcations.

**Reference**

1. Kurihara T, Shimizu-Hirota R, Shimoda M, Adachi T, Shimizu H, Weiss SJ, Itoh H, Hori S, Aikawa N, Okada Y: **Neutrophil-derived matrix metalloproteinase 9 triggers acute aortic dissection.** *Circulation* 2012, **126:**3070-3080.

2. Yao F, Yao Z, Zhong T, Zhang J, Wang T, Zhang B, He Q, Ding L, Yang B: **Imatinib prevents elastase-induced abdominal aortic aneurysm progression by regulating macrophage-derived MMP9.** *Eur J Pharmacol* 2019, **860:**172559.
